# The McGill University Department of Oncology: structure depicts the shape of evolving knowledge

**DOI:** 10.3747/co.v15i3.214

**Published:** 2008-06

**Authors:** G. Batist, G.A. Shinder

**Affiliations:** * Department of Oncology, McGill University, Gerald Bronfman Centre for Clinical Research in Oncology, Montreal, QC

**Keywords:** Interdisciplinary programs, basic research, translational research, clinical research, clinical practice

## Abstract

The McGill University Department of Oncology has changed and expanded since its inception in 1990, responding to the move to interdisciplinary clinical care, teaching, and research. Although the traditional Divisions have been maintained to correspond to University and Royal College interfaces, the department has steadily been generating a variety of cross-departmental and interdisciplinary programs in which new insights into clinical care and biology are being generated. In research areas ranging from psychosocial and fundamental to translational and clinical therapeutics, interdisciplinarity and an emphasis on clinician–scientists are critical features.

## 1. HISTORICAL INTRODUCTION

The McGill University Department of Oncology was founded in 1990 by then Dean of Medicine, Dr. Richard Creuss. It was the first such department in Canada, having been made possible by a generous donation from the Bronfman family in memory of Minda de Gunzburg, daughter of Samuel and Saidye Bronfman and wife of Baron Alain de Gunzburg. Dr. Brian Leyland–Jones chaired the Department from 1990 to 2000 as the first Minda de Gunzberg Professor.

A second generous donation from Marjorie and Gerald Bronfman allowed clinical research in oncology at McGill to flourish, and in May 1992, the Gerald Bronfman Centre for Clinical Research in Oncology officially opened its doors. That building is currently the home base for the Department of Oncology and the location of the department’s administrative offices. Other departmental programs currently headquartered in the building include the centralized Clinical Research Program, the Division of Cancer Epidemiology, Cancer Genetics, the Cancer Nutrition-Rehabilitation program, and the McGill Programs in Whole Person Care.

Other endowments that were established in the early days of the department include the Helen and Sam Steinberg Family Career Award and the Flanders Chair in Palliative Care.

The Department of Oncology was formed at a time when, as nearly everywhere in Canada, clinical oncology was largely invested in a department of radiation therapy while medical oncology was either a subunit of hematology or else a standalone division within a department of medicine. The McGill Cancer Centre (mcc), originally founded by Dean Samuel Freedman under the directorship of Dr. Phil Gold, was a grouping of affiliated research laboratories at the Medical Faculty Building. In time, the mcc developed a clinical research program under the leadership of Dr. Richard Margolese. With his great success in the National Surgical Adjuvant Breast and Bowel Project, he joined with other clinical leaders—Drs. Henry Shibata and Lawrence Hutchison, among them—to generate successful Cancer and Leukemia Group B funding and to initiate participation in the National Cancer Institute of Canada (ncic) at a time when little investigator-initiated clinical research was occurring.

The new Department of Oncology had a classical divisional structure in which the erstwhile Department of Radiation Oncology nobly accepted its role as a division within the department, the mcc became the Division of Basic Cancer Research, and mcc Clinical Research was recognized as the new Division of Clinical Oncology. To these were added the divisions of Epidemiology and of Palliative Care. However, right from the start, it was evident that modifications were required. Within the first 18 months, “Clinical Trials Operations” was established and a Pediatric Oncology division was created, as was a transdivisional section of Experimental Therapeutics, with the goal of generating a greater number of investigator-initiated trials.

Further changes followed, such that by 1995, the Division of Clinical Oncology was split into the divisions of Medical Oncology and of Surgical Oncology. In addition, 13 trans-hospital multidisciplinary research sections were created to develop, review, and oversee clinical research activities for various tumour types—for example, leukemia, lymphoma, melanoma, neuro-oncology, endocrine/biologics, breast cancer, and so on. The department encompassed research and training at the Montreal General Hospital, The Royal Victoria Hospital, St. Mary’s Hospital, and the Sir Mortimer B. Davis–Jewish General Hospital. In addition to Clinical Trials Operations at the Gerald Bronfman Centre, the early 1990s saw the creation of the Clinical Research Unit at the Sir Mortimer B. Davis–Jewish General Hospital, the focal point for phase i and complex phase ii clinical trials.

As clinical care and research in oncology were being structured and expanded, the department also gave top priority to oncology teaching and training. By the mid-1990s, a Medical Education Committee to address undergraduate education and four oncology residency training programs (medical, radiation, surgical, and hematologic oncology) were established. Furthermore, a new lecture series, the bi-annual Cedars Visiting Professorships was created in the early 1990s to supplement Oncology Grand Rounds. Over the years, that lecture series changed and evolved to become the Visiting Speakers in Oncology program. The latter program, a joint effort with the Université de Montréal, sponsors approximately 20 visiting professors annually.

## 2. ONCOLOGY IN THE NEW MILLENNIUM: INTERDISCIPLINARY TEAM-BUILDING

Starting in the late 1990s, it became increasingly clear that a much closer link was essential between scientists and health care professionals in all fields to capitalize on scientific advances in cancer control. Not only is new knowledge required, but also the ability to translate biologic knowledge into clinically relevant questions or even therapies, and then to transfer new clinical knowledge to caregivers, the public, and health policymakers. Optimal conditions for everything from the development of innovative therapies to the training and continuing education of professionals requires an intimate linking of efforts from various medical disciplines—and ultimately from various health care professions. Furthermore, the University has recently been called on to play a larger role in the planning and delivery of health care outside of its traditional institutional boundaries.

### 2.1 Réseau universitaire intégré de santé

Quebec has established a novel structure of health care based on the four university-based health networks (McGill University, Université de Montréal, Université de Sherbrooke, and Université de Laval), each one servicing a particular geographic region and responsible for the coordination of health care services, research, and teaching in its area. The McGill Department of Oncology is the central hub for coordinating oncology care, research, and education within the McGill Réseau universitaire intégré de santé (ruis) region, and it plays a critical role in integrating those activities within the McGill-affiliated institutions [McGill University Health Centre (muhc), Segal Cancer Centre–Sir Mortimer B. Davis–Jewish General Hospital, St. Mary’s Hospital Centre]. The “table d’oncologie,” a committee of representatives from all of the foregoing institutions and of various disciplines of cancer care, plus epidemiologists, health care administrators, pharmacists, and patient representatives, is the coordinating body for the department.

The table d’oncologie meets monthly and implements programs of outreach to remote communities, video and telephone consultations, rapid inter-institutional referrals, practice guidelines, and policies. A guiding principle is ready access to the standard of practice as close to home as possible. Research collaborations are also developing between the university centres and the regional authorities. For example, evidence accumulated by the Abitibi–Témiscamingue public health authorities showed that some water and soil may contain significantly elevated levels of arsenic, undoubtedly related to the region’s history of mining. As it happens, Dr. Koren Mann at the Segal Cancer Centre has a developing interest and expertise in the environmental aspects of arsenic and is investigating this problem in areas of the U.S. Southwest, just as her associate Dr. Wilson Miller is an authority on arsenic’s emerging use as a cancer therapeutic. Such obvious opportunities for research programs that engage university-based scientists with remote regions are being exploited by the ruis relationship.

### 2.2 Interdisciplinary Programs

**FIGURE 1:**
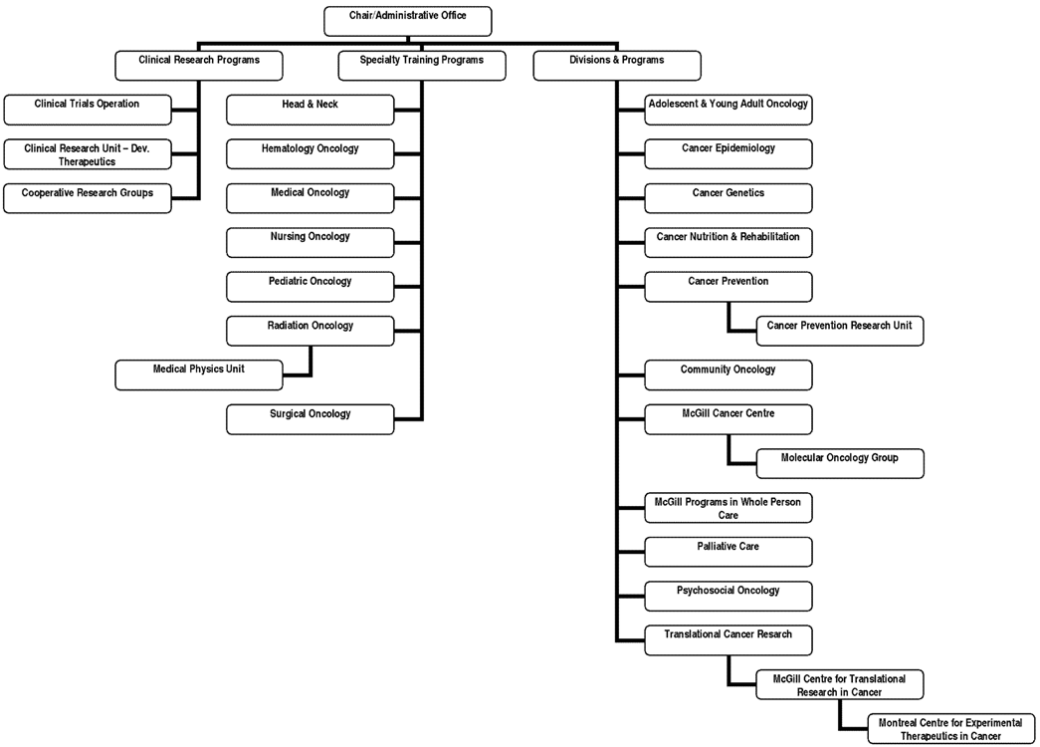
Department of Oncology, McGill University.

In response to the evolving nature of the relationship between fundamental knowledge discovery and translational research, and subsequent knowledge transfer into clinical practice (where population-based studies are then employed), the McGill Department of Oncology has changed its shape and internal configuration. The aim is to further the goal of an integrated approach to cancer care, research, and education by creating a number of interdisciplinary programs that transcend disease sites and specific tumour types, concentrating instead on specific areas of research and treatment (Figure 1). The classical divisions remain, mostly to provide a context for the various training programs and to interface with professional organizations that are based on subspecialties, but the activities of the members of each of the interdisciplinary groupings have clearly breached the classical division boundaries.

For example, each of the McGill-affiliated hospitals has a team of medical oncologists, some of whom provide services at more than one hospital, and many of whom regularly link by videoconference for various meetings to facilitate networking and sharing of ideas. In addition to being on the front line in diagnosing and treating cancer patients, some of the medical oncologists are also involved in other aspects of cancer care such as screening, prevention, rehabilitation, symptom management, and palliative care. The medical oncologists work in interdisciplinary teams with surgical and radiation oncologists, nurses, and oncology pharmacists to provide the best possible care for their patients. They also are actively involved in many clinical studies, thus giving their patients access to the most recent advances in cancer therapy. A number of the medical oncologists also have productive and well-funded fundamental and translational research programs.

Similar developments have also occurred in hematology–oncology and in surgical oncology, where research programs such proteomic and genomic array technologies for biomarker discovery and validation, conducted by clinician–scientist Dr. Mark Basik, clearly blur the distinction between disciplines. The Division of Radiation Oncology, directed by Dr. Carolyn Freeman, is already multidisciplinary, comprising physicians, physicists, and radiation scientists. That division is well known for its technology development and clinical application of innovations in medical physics. Dr. Ervin Podgorsak has been a remarkable innovator, providing the groundwork for a variety of therapeutic approaches in a range of tumour types from brain irradiation to endo-rectal high dose rate brachytherapy.

For the most part, however, the current structure of the department reflects the multi- and interdisciplinary nature of both clinical practice and scientific inquiry that is the emerging model. In fact, it reflects that model to the point that the department’s training program has been highlighted by the creation of an Interdisciplinary Training Committee designed to foster inter-professional interactions among the clinical trainees as preparation and prelude to the careers that they face in the near future. The Division of Cancer Epidemiology (Dr. Eduardo Franco) played a key role through a regular journal club attended by the trainees from all disciplines.

An innovative example of transcending departmental structure was the development and recent university approval of the Psychosocial Oncology Option, which is coordinated through the Department of Oncology and forges links between faculty and students in the Departments of Oncology and Psychology and in the Schools of Nursing and Social Work. The Psychosocial Oncology Option is further discussed under the Psychosocial Oncology Program later in this article.

#### 2.2.1 Division of Cancer Epidemiology

A generous endowment to McGill in 1988 by the Cancer Research Society resulted in the establishment of the Division of Cancer Epidemiology. Although primarily a unit within the Department of Oncology, Cancer Epidemiology, which is directed by Dr. Eduardo Franco, is also part of the McGill Department of Epidemiology and Biostatistics. The program conducts epidemiologic research on the causes and prognosis of cancers, with the aim of providing recommendations for prevention strategies. Its main foci are cervical cancer, upper aero-digestive tract tumours, and childhood malignancies. Its research program in cervical cancer has examined the natural history of cervical cancer, precursor lesions, and also risk determinants such as infection with the human papillomavirus (hpv). Researchers in the program have also investigated the epidemiology of environmental and social determinants of upper aero-digestive tract cancer and childhood cancers.

In the area of cancer prevention, researchers in Cancer Epidemiology have also been developing mathematical models to assess the cost-effectiveness of cancer prevention strategies in Canada and internationally. That research has been carried out internationally with studies and collaborations in Brazil, the Republic of Congo, and Lyon, France (with the International Agency for Research on Cancer).

Cancer Epidemiology also offers its expertise in statistical and study planning to other researchers in the Department of Oncology, and it has an active teaching program for undergraduate and graduate students and postdoctoral and medical fellows.

#### 2.2.2 Division of Palliative Care

The Division of Palliative Care, directed by Dr. Anna Towers, includes members from the various McGill-affiliated institutions. The Division not only provides clinical care to adult cancer patients in the final stages of their disease, but also to pediatric palliative care patients through specially trained physicians based at the Montreal Children’s Hospital. Palliative Care Research, directed by Dr. Robin Cohen, is an important component of the Division and many of its members conduct research programs in areas such as pain control, lymphedema, anorexia–cachexia, and quality of life. Dr. Cohen also directs the Canadian Institutes of Health Research (cihr)/ncic Strategic Training Program in Palliative Care, which is a collaborative training program involving McGill, Université de Laval, and the University of Ottawa. Consistent with the general move toward integration and transcendence of disciplinary barriers, Dr. Bernard Lapointe’s Palliative Care Program at the Jewish General Hospital was designated an “integrated program” in 2007, the first North American site to be so designated by the European Society of Medical Oncology. Dr. Lapointe’s vision has moved palliative care into the “supportive care” realm, focusing not only on end-of-life care, but also on symptom management, both medical and psychosocial, of curable patients early in their disease trajectory. This expansion of the boundaries of the discipline has been taken up by other McGill palliative care doctors, including Drs. Neil MacDonald and Martin Chasen, who work on the nutritional and rehabilitation aspects. Some of Dr. Cohen’s research even departs from direct contact with the patient to focus on family caregivers, a move that is strikingly insightful and responsive to a real need in today’s society.

#### 2.2.3 Other Interdisciplinary Programs

##### The McGill Head and Neck Cancer Program

Directed by Dr. Martin Black, this group functions in two hospitals, handling high-quality care, clinical research, and teaching. Research activities include novel therapeutics and tumour biomarkers (Dr. Moulay Alaoui–Jamali). The group also has a strong link to the McGill School of Dentistry, where Dr. Paul Allison leads a large program that touches on public and oral health, and the psychosocial aspects of head and neck cancer. In addition, Dr. Franco has generated a significant research program based initially on the role of hpv virus in nasopharyngeal cancer.

##### The McGill Pediatric Oncology Program

Centred at the Montreal Children’s Hospital, the Pediatric Oncology Program provides care for cancer patients 0–18 years of age. The hospital’s pediatric neuro-oncology program, with its interdisciplinary team that includes 3 skilled pediatric neurosurgeons, sees 65% of pediatric brain tumours in Quebec. Dr. Nada Jabado is a clinician–scientist with a major translational research program in brain tumours. The Montreal Children’s Hospital is also the focal point for the Quebec retino-blastoma program, collaborating with staff from Hôpital Ste. Justine. Most of the services necessary for care provision are available on site, but the pediatric oncology team at the Montreal Children’s Hospital also works collaboratively with the adult hospitals to provide services such as radiotherapy, some laboratory tests, a long-term follow-up clinic, and the Adolescent and Young Adult Oncology Program.

The McGill Pediatric Oncology Program is actively involved in clinical research as a full member of the Children’s Oncology Group, and provides training to postgraduates and fellows enrolled in the general surgery, neurosurgery, and radiation oncology programs.

##### The Adolescent and Young Adult Oncology Program

Caught between childhood and adulthood, adolescents and young adults with cancer must not only contend with the unique challenges of their stage in life, but also with the cancer experience itself. Established in early 2003 under the leadership of Dr. Petr Kavan, the Adolescent and Young Adult Oncology Program deals with the different types of cancers that occur in young adults as compared with adults, and that tend not to respond as well to adult treatment protocols. The goals of the program are to provide optimal interdisciplinary care for this unique population; to involve the patient population more actively in national and international multicentre research protocols and projects, and in in-house protocols; to improve the access of this population to psychosocial support services (social services, psychologists, psychiatrists); and to improve teaching about and research into the challenges faced by adolescent and young adult cancer patients.

##### The Cancer Prevention Program

Under the direction of Dr. Michael Pollak, the Cancer Prevention Program (www.mcgill.ca/cancerprev) was inaugurated in October 2002. It includes an academic research program and clinical and educational programs designed to make the public aware of cancer prevention. Clinical programs are based at the Stroll Family Cancer Prevention Centre at the Segal Cancer Centre of the Sir Mortimer B. Davis–Jewish General Hospital. The Centre offers programs in smoking cessation, diet, and lifestyle, and also coordinates the activities at the Segal Cancer Centre of the cancer geneticists who work with people concerned about the possibility of genetic predisposition to cancer in their family. The Cancer Prevention Program’s research component comprises an interdisciplinary group of researchers and clinical specialists who conduct laboratory and epidemiologic research projects aimed at improving the understanding of cancer risk and ultimately preventing cancer. Members of the program team deliver lectures to undergraduates and medical students and provide opportunities for medical students to carry out electives in cancer prevention. In addition, members of the team provide research projects for graduate students and postdoctoral fellows interested in cancer risk and cancer prevention.

##### McGill Program in Cancer Genetics

The Cancer Genetics Program (www.mcgill.ca/cancergenetics), under the direction of Dr. William Foulkes, was established as a joint program of the departments of Oncology and of Human Genetics. The goal was to establish, in Montreal, a leading interdisciplinary centre for hereditary cancer genetics that is an ideal context for clinical service, research, and teaching. Together with colleagues in Pathology at the Sir Mortimer B. Davis–Jewish General Hospital, the Program in Cancer Genetics is active in several areas of hereditary cancer, in particular hereditary breast and ovarian cancer caused by mutations in *BRCA1* and *BRCA2,* and hereditary colorectal cancer. In the past year, Dr. Marc Tischkowitz and colleagues have focused on determining the contribution of a new breast cancer gene, *PALB2,* to the incidence of breast cancer in Quebec. Dr. Foulkes and colleagues identified and characterized a novel mutation in *PMS2* (a hereditary colorectal cancer gene) that is present in at least two families of Inuit origin from Nunavik (unpublished data). The identification of the same mutation in two unrelated families could have consequences for screening in that remote population. Substantial work has also been carried out by Dr. Patricia Tonin with respect to ovarian cancer pathogenesis. Recently, Dr. Raquel Aloyz and colleagues have begun to look at cell line models of cancer and how new drugs may be effective against hereditary forms of breast cancer (unpublished data). In terms of international visibility, the Program hosted, in collaboration with the Hereditary Breast and Ovarian Cancer Foundation (www.hboc.ca), the Second International Symposium on Hereditary Breast and Ovarian Cancer, Montreal, October 17–19, 2007. More than 250 attendees listened to 30 international, national, provincial, and local researchers present a comprehensive overview of hereditary breast and ovarian cancer, from the base pairs altered in individuals at risk to the psychological dimension of being a gene carrier.

##### The McGill Cancer Nutrition-Rehabilitation Program

The McGill Cancer Nutrition-Rehabilitation (cnr) Program (www.mcgill.ca/cnr), whose founding director is Dr. Neil MacDonald, was instituted in 2002 to treat cancer patients experiencing poor appetite, malnutrition, weight loss, fatigue, and loss of function. The Program has two clinic sites, one at the Segal Cancer Centre of the Sir Mortimer B. Davis–Jewish General Hospital (Medical Director: Dr. MacDonald) and one at the muhc–Royal Victoria Hospital (Medical Director: Dr. Martin Chasen). The administrative office is housed at the Gerald Bronfman Centre at McGill.

The mission of the McGill cnr Program, which encompasses clinical care, research, and education, is to develop and administer nutritional, rehabilitation, and psychosocial programs for cancer patients who are suffering from poor appetite, malnutrition, weight loss, fatigue, loss of function, and psychological distress; to conduct research aimed at understanding the underlying biologic reasons that patients with cancer suffer from the foregoing symptoms; to educate patients, family caregivers, and health professionals in new ways to manage those symptoms; to take part in national and international study groups that investigate new medical treatments and other therapies, and to enrol program patients into ethically approved trials; to investigate and provide for the needs of patients who are “cancer survivors”; and to act as a referral and reference centre for patients and professionals.

The dedicated team of professionals in the program include physicians specializing in cancer nutrition, rehabilitation, anticancer therapies, and symptom control; nurses who are experienced in providing health care, education, and support to cancer patients and their families; physiotherapists who can design individualized exercise programs for patients to improve their physical performance and stamina; dietitians whose chief aims are to enhance and optimize the nutritional intake of patients, allowing for optimal weight; occupational therapists who specialize in functional assessment and implementation of activity-directed rehabilitative programs, thus aiding patients to function optimally in the activities of daily life; and a psychologist with experience working with patients throughout the illness trajectory. In October 2007, Dr. Thomas Jagoe, a pulmonologist whose research interests include mechanisms of muscle wasting, particularly in cancer, joined the group as Program Director.

##### The Psychosocial Oncology Program

The Psychosocial Oncology Program was initially a joint venture of the McGill departments of Oncology and of Psychiatry. The program is currently directed by Dr. Zeev Rosberger and incorporates clinical psychology, psychiatry, nursing, palliative care, and quality of life research. The main goals of the program are to develop research capacity and enhance recruitment of psychosocial oncology researchers; to train and supervise graduate students; and to develop a teaching program in psychosocial oncology research. To further the latter goal, the Department of Oncology has forged links with the Department of Psychology, the School of Nursing, and the School of Social Work to initiate a new program option called the Psychosocial Oncology Option. Doctoral students in the School of Nursing and the Department of Psychology who are interested in psychosocial oncology can take this program option. Students are required to take Psychosocial Oncology Research, a course given by the School of Nursing’s Dr. Carmen Loiselle, who is an Associate Member of the Department of Oncology, and the course Palliative Care in Cancer given by Dr. Cohen from the Department of Oncology. In addition, they are required to choose one of five complementary courses given by the Department of Psychology or the School of Social Work. This program option provides an excellent opportunity for participating students and academic staff in various disciplines to join forces and generate new psychosocial oncology knowledge that may ultimately be used in clinical settings.

##### Nursing Oncology

The Department of Oncology recognizes the central role that nursing plays in cancer care and has worked closely with the School of Nursing and an established group of academic nurses whose research interests focus on oncology nursing. The clinical research programs are aimed at providing a better understanding of the psychosocial issues faced by cancer patients and their families, including emotional stress, decision-making, and information-seeking strategies. Dr. Carmen Loiselle is the director of the program and links it with nursing colleagues throughout McGill and beyond. The recent introduction of “nurse navigators in oncology” (“infirmières pivots en oncologie”) in Quebec provides an important opportunity for Dr. Loiselle and her colleagues to critically evaluate various models for this role.

In 2003, the Psychosocial Oncology Research Training program was created as a result of a Strategic Training Grant awarded to Dr. Loiselle and her colleagues by the cihr–Institute of Cancer Research and ncic. The program comprises a multidisciplinary team of researchers (nursing, psychology, management, and medicine) from McGill University, the University of Manitoba, the University of British Columbia, and Dalhousie University, and was designed to provide graduate-level fellowships and awards so that young researchers can learn to develop, test, and refine psychosocial cancer care interventions that are effective, accessible, and beneficial for people dealing with a cancer diagnosis.

##### The McGill Programs in Whole Person Care

The McGill Programs in Whole Person Care, under the direction of Dr. Thomas Hutchinson (as of 2004), were instituted in February 1999 on the initiative of Drs. Balfour Mount and Abraham Fuks (the latter being Dean of Medicine at the time). The goal of the programs is to better understand how to respond to suffering experienced by the whole person by incorporating the physical, psychosocial, and existential/spiritual aspects of the person. The idea behind this approach is to allow for healing in situations in which treatment is unlikely to change the outcome of the disease.

Research programs use qualitative methodology to focus on determinants of healing and whole-person care. Members of the multidisciplinary team have expertise in qualitative methodology, mindfulness-based stress reduction, end-stage renal disease, palliative care, and healing. Members teach undergraduate- and graduate-level courses and are responsible for the healing component of the Physicianship Program in the Faculty of Medicine. Two public education programs are also offered on a monthly basis:

- Invited speakers give presentations at the lunch-time series McGill Seminars in Healing
- The series Films That Transform uses film and follow-up dialogue with the series discussants to help the audience achieve a better understanding of healing in a biomedical context

##### The Community Cancer Care Program

Under the direction of Dr. Jaroslav Prchal at St. Mary’s Hospital Centre, the mandate of the Community Cancer Care Program is to encourage the development of cancer care in community hospitals so that patients can be treated as close to their living environment as possible. To provide the best service to the ever-growing numbers of cancer patients and cancer survivors, raising the number of physicians trained in cancer care is of utmost importance. To achieve that goal, the Community Cancer Care Program plans to provide specific training sessions to general practitioners and family practitioners and to provide increased cancer treatment education at the undergraduate and postgraduate levels.

### 2.3 Cancer Research

#### 2.3.1 McGill Cancer Centre

The mcc, established in 1978, is the core of basic cancer research at McGill, coordinating cancer research activities within various McGill departments and affiliated hospitals. Under the direction of Dr. Michel Tremblay, the mcc’s research activities focus on a wide range of topics such as signal transduction, differentiation, cell proliferation, gene therapy, radiation resistance, and drug development. The mcc has recently linked with the Molecular Oncology Group, which until recently was based at the Royal Victoria Hospital. It was established in 1994 as an independent research division within the Department of Medicine and is internationally renowned for its work on cellular and molecular biology of cancer. The group, headed by Dr. Morag Park, comprises six laboratories whose research programs focus on the molecular control of cell growth and differentiation and the effect of changes at the genomic or proteomic level on tumour initiation and progression. Collaborations with medical and surgical oncologists, pathologists, and pharmaceutical and biotechnology companies facilitate the translation of research findings into therapeutic strategies and drug development.

#### 2.3.2 McGill Centre for Translational Research in Cancer/Montreal Centre for Experimental Therapeutics in Cancer

The Montreal Centre for Experimental Therapeutics in Cancer (mcetc) was established in 2000 as a result of a Canada Foundation for Innovation (cfi)/Fonds de la recherche en santé du Québec (frsq) infrastructure award to the McGill Centre for Translational Research in Cancer (mctrc) for the construction of a state-of-the-art laboratory that would provide a link between basic and clinical research. With the addition of major infrastructure funding from Quebec and the successful recruitment of new members to the Centre, the facility that was built is twice as large as originally planned. The new facility, which was up and running by October 2005, occupies the fourth and fifth floors (more than 3700 m^2^) of the newly built E-wing expansion at the Sir Mortimer B. Davis–Jewish General Hospital. It is closely linked with the Segal Cancer Centre on the seventh through ninth floors of the expanded E-wing.

The mctrc/mcetc has research programs covering four main themes: drug target discovery and validation, new drug development, cell and gene therapy, and prevention and risk assessment. The Centre also provides eight core facilities: a clinical research unit, cell preparation centre, dna and tissue bank, viral vector production unit, flow cytometry unit, high-performance computational biology and drug development unit, peptide synthesis laboratory, and research pathology core facility. The centre, with its high throughput system of both preclinical and early-phase clinical trials, has made major contributions to the development of novel therapies.

In addition to the research being performed at the newly built facility, the mctrc/mcetc team also includes researchers at four other universities—the Université de Montréal, Université du Québec à Montréal, Université de Laval, and Université de Sherbrooke—and six hospital-based or independent research institutes—Lady Davis Institute for Medical Research, the Institut national de recherche scientifique–Institut Armand Frappier, Ste. Justine Hospital Research Centre, Biotechnology Research Institute of the National Research Council, Centre de recherche clinique et évaluative en oncologie du Centre hospitalier universitaire de Québec (crceo-chuq), and Centre hospitalier universitaire de Sherbrooke.

#### 2.3.3 Clinical Research

The Clinical Research Program in the Department of Oncology is co-directed by Drs. Wilson Miller from the Sir Mortimer B. Davis–Jewish General Hospital and Catalin Mihalcioiu from the muhc–Montreal General Hospital. The program oversees phase i, ii, and iii oncology clinical trials (sponsored by ncic, the Radiation Therapy Oncology Group, pharmaceutical and biotechnology companies) and investigator-initiated studies that are conducted at McGill-affiliated hospitals.

The Clinical Research Unit of the Sir Mortimer B. Davis–Jewish General Hospital, which was established as the first of its kind in Quebec in 1993, moved in early 2006 to its new location on the eighth floor of the Segal Cancer Centre. The unit is directed by Dr. Miller and conducts phase i and early phase ii oncology clinical trials with a strong focus on hematologic oncology. The treatment area includes four beds, three chemotherapy treatment chairs, and an additional single patient room. Patients come not only from McGill-affiliated hospitals, but also from hospitals throughout Quebec. The Unit is an integral part of the Montreal Centre for Experimental Therapeutics in Cancer and its goal to bring cancer research from the laboratory bench to the clinic.

## 3. FUTURE DIRECTIONS

### 3.1 New Program

Ever-alert for joint ventures that cross disciplinary lines, the Department of Oncology has launched a project to develop a Cancer Pathology Program that will link its work with that of the Department of Pathology. The emerging central role of molecular pathology, both for research and for individualized clinical care, makes that link imperative.

### 3.2 Networking Across Quebec

The two cfi-sponsored cancer centres in Quebec, the Segal Cancer Centre at the Jewish General Hospital and the Centre de recherche clinique et évaluative en oncologie (crceo) of the Hôtel-Dieu du Québec (Director: Dr. Luc Bélanger) have enjoyed a natural link since they cross-referenced one another in their original applications, and they since have shared major funding. Together, they are the experimental therapeutics axis of the frsq’s Cancer Research Network (Directrice: Dr. Anne-Marie Mes–Masson). They have now linked to form the Quebec Clinical Research Organization in Cancer, whose initial major program is profiling clinical resistance. The program garnered the active participation of major clinical scientists and basic researchers from McGill, Université de Montréal, Université de Sherbrooke, and Université de Laval. The hope is that this collaboration will bring energy and structure to an evolving clinical and translational research network in Quebec.

## 4. SUMMARY

As knowledge becomes both more complex and more useable, it necessarily crosses established disciplinary and university departmental boundaries. This boundary crossing has resulted in novel insights and remarkable synergies. The associated change is not easy; it requires a rethink of many of the structures upon which budgets and career planning have generally been made. Yet it is an irresistible movement that the McGill Department of Oncology has tried to join and to incorporate into its development. It includes delivery of health care, research, and teaching. The training aspect is most important, because the rate of change is so rapid that continuous revisions and rethinking are required.

